# Determination of synthetic hallucinogens in oral fluids by microextraction by packed sorbent and liquid chromatography–tandem mass spectrometry

**DOI:** 10.1007/s00216-023-04751-2

**Published:** 2023-05-23

**Authors:** Evan Lesne, Miguel Muñoz-Bartual, Francesc A. Esteve-Turrillas

**Affiliations:** Department of Analytical Chemistry, University of Valencia, 50th Dr. Moliner St., 46100 Burjassot, Spain

**Keywords:** New psychoactive substances, NBOMes, Oral fluids, Microextraction by packed sorbent, Liquid chromatography–tandem mass spectrometry

## Abstract

A fast and simple procedure based on microextraction by packed sorbent (MEPS) and liquid chromatography–tandem mass spectrometry (LC–MS/MS) has been developed for the simultaneous quantification of 28 synthetic hallucinogens in oral fluids, including lysergic acid diethylamide and substances from NBOMe, NBOH, NBF, 2C, and substituted amphetamine categories. Extraction conditions such as type of sorbent, sample pH, number of charge/discharge cycles, and elution volume were studied. Hallucinogenic compounds were extracted from oral fluid samples using C18 MEPS, loading with 100 μL sample (adjusted to pH 7) in 3 cycles, washing with 100 μL deionized water, and eluting with 50 μL methanol in 1 cycle, giving quantitative recoveries and no significant matrix effects. Limits of detection from 0.09 to 1.22 μg L^−1^; recoveries from 80 to 129% performed in spiked oral fluid samples at 20, 50, and 100 μg L^−1^; and high precision with relative standard deviations lower than 9% were obtained. The proposed methodology was demonstrated to be appropriate for the simple and sensitive determination of NBOMe derivates and other synthetic hallucinogenic substances in oral fluid samples.

**Graphical Abstract:**

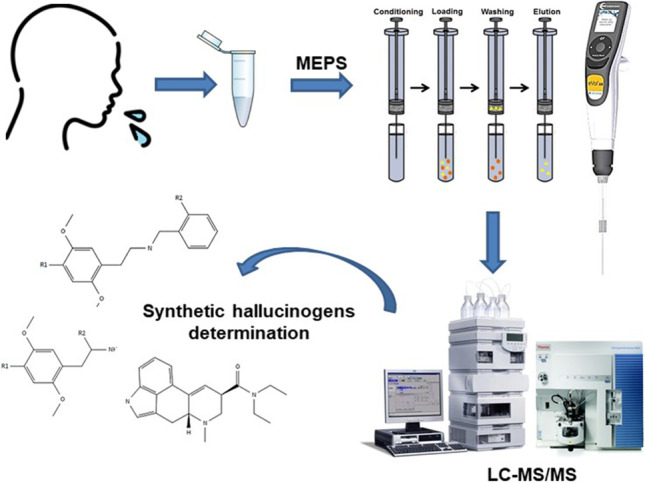

## Introduction

New psychoactive substances (NPS) refer to a category of substances in a pure form or in the form of a preparation, which are not controlled under the Single Convention on Narcotic Drugs of 1961 or the Convention on Psychotropic Substances of 1971, but may pose a threat to public health. These substances can be analogues of existing controlled drugs or newly synthesized chemicals designed to mimic the psychoactive effects of controlled drugs. The number of NPS identified by national authorities and forensic laboratories over the last 15 years totalled 1.127 by December 2021. This is more than triple the 302 psychoactive substances under international control at the end of 2021 [1]. The main problem of NPS is that many emerge constantly for only a short period of time before disappearing, and their toxicity, psychoactive effects, potency, and risk are generally unknown. Monitoring NPS is a complex challenge because NPS are a fluid category that includes a large group of substances.

The 2021 European Drug Report from the European Monitoring Centre for Drugs and Drug Addiction highlights that hallucinogenic substances are widely available in Europe, but they are poorly monitored. Thus, more information is required to evaluate the real impact of hallucinogens on public health [2]. Among synthetic hallucinogens, NBOMe-type substances have gained a high notoriety in the past years. NBOMes are *N*-benzylmethoxy derivatives of the 2C family of hallucinogens which acts as a very potent 5-HT_2A_ receptor agonist [3]. These drugs are designed to mimic the effects of conventional hallucinogens like lysergic acid diethylamide (LSD) and mescaline, the NBOMe dosage levels in humans being similar to those of other hallucinogens [4]. NBOMe derivatives are usually consumed sublingually via blotter paper, and they are often falsely sold as lysergic acid [5, 6]. Since their introduction into the drug market, NBOMe derivatives have been frequently associated with intoxication and fatal incidents appearing in reported cases, such as 25B-NBOMe [7, 8], 25I-NBOMe [8], 25C-NBOMe [9], and 25B-NBOH [10]. Thus, the abuse of NBOMes and other derivatives like NBOH and NBF-type substances presents a severe risk to consumers, the fast and correct identification of the consumed hallucinogen being crucial to provide an effective intoxication treatment.

New synthetic hallucinogens show a high potency, with doses being much lower than the classical ones. The amount found in seized blotters typically ranges from 0.01 to 0.10 mg for LSD, from 0.5 to 4.2 mg for DOC, and from 0.5 to 1.5 mg for NBOMes [5, 11]. Thus, the concentrations of hallucinogens in biological fluids are very low, with found concentrations between 0.18 and 2.8 µg L^−1^ in serum and blood samples [12] and reaching very high values, from 38 to 661 µg L^−1^, in the case of severe intoxications. In the case of urine samples, NBOMe concentrations are in the 0.9–2.8 µg L^−1^ concentration range [13]. However, to our knowledge, there are no studies in the literature that estimate any correlation between blood/urine or blood/oral fluid concentration levels of synthetic hallucinogens. Thus, the analysis of biological samples requires an efficient extraction approach to eliminate matrix interferents and to preconcentrate analytes, as well as a very highly sensitive and selective technique to provide an unambiguous determination of the abuse substance. Analytical procedures developed for the identification and quantification of novel hallucinogens are mainly focussed on the analysis of matrices such as blotter paper [14, 15] and several biological fluids like plasma [16], blood [12], hair [17], and urine [18].

Oral fluid is an emerging biological matrix that has gained a high acceptance as an alternative biological matrix for detecting illicit drugs in forensic and clinical areas, due to its easy sample collection through non-invasive procedures [19]. However, there are scarce studies involved in the analysis of a few number of NBOMe-type compounds in oral fluids, which are determined by different techniques like liquid chromatography–tandem mass spectrometry (LC–MS/MS) [20–22] and differential pulse voltammetry [23].

Regarding the extraction approach, the most widely employed techniques for the extraction of psychoactive substances in biological fluids are liquid–liquid extraction (LLE) and solid-phase extraction (SPE) [24, 25]. Nevertheless, microextraction techniques offer higher advantages than those offered by conventional techniques, such as reduced reagent consumption, short extraction time, minimum required volume of sample, potential automation, and reduced environmental impact. Among these techniques, microextraction by packed sorbent (MEPS) is one of the most promising approaches [26]. MEPS is a miniaturized version of SPE, where a few milligrams of sorbent are packed at the end of a needle that is inserted in a semiautomatic syringe [27]. This miniaturization greatly reduces the required sample and eluent volumes from the millilitre to the microlitre range, being of the same magnitude to be injected into chromatographic systems, and then increasing sensitivity. Moreover, MEPS is commercially available, can be fully automated, and is appropriate for the rapid analysis of biological samples. The use of MEPS is of particular interest for the analysis of psychoactive substances in clinical and forensic areas, and it has been employed for the extraction of cocaine and its metabolites from hair samples [28]; opiates from whole blood [29]; drugs of abuse and synthetic cathinones [30] and designer benzodiazepines and Z-hypnotics [31] from plasma; ketamine and norketamine [32], epinephrine derivatives [33], and tryptamines [34] in urine; and assorted NPS [35, 36], synthetic cannabinoids [37, 38], methylone [39], and dichloropane [40] in oral fluids.

The aim of this work is to develop a fast, simple, and sensitive procedure for the determination of 28 synthetic hallucinogenic substances in oral fluids based on MEPS and LC–MS/MS. The studied analytes involved LSD, 16 NBOMes, 3 NBOHs, 3 NBFs, 3 2-C compounds, and 2 substituted amphetamines. Extraction conditions for MEPS have been selected to provide quantitative recoveries, and the analytical performance of the method has been assessed in terms of linearity, sensitivity, precision, accuracy, and matrix effect.

## Experimental procedure

### Experimental apparatus and reagents

Stock solutions of the studied synthetic hallucinogens (Table 1) were provided by Cayman (Ann Arbor, MI, USA). Deuterated LSD-D3 and 25C-NBOMe-D3, provided by Sigma-Aldrich (Steinheim, Germany), were employed as internal standards. Standard working solutions were prepared in methanol and stored at − 18 °C in amber glass vials.

**Table 1:** Instrumental parameters for analytes under investigation

Analyte	CAS number	Formula	RT (min)	MRM_1_ (m/z)	CE_1_ (eV)	MRM_2_ (m/z)	CE_2_ (eV)	IS
2C-C	88441–14-9	C_10_H_14_ClNO_2_	1.47	216.1 > 198.9	11	216.1 > 184.2	19	LSD D3
DOC	123431–31-2	C_11_H_16_ClNO_2_	1.80	229.9 > 213.3	10	229.9 > 185.2	17	LSD D3
2C-E	71539–34-9	C_12_H_19_NO_2_	2.11	209.9 > 193.3	11	229.9 > 178.3	18	LSD D3
LSD	50–37-3	C_20_H_25_N_3_O	2.33	324.2 > 223.2	23	324.2 > 207.2	45	LSD D3
LSD D3 (IS)	136765–38-3	C_20_H_22_D_3_N_3_O	2.34	327.0 > 226.2	24	327.0 > 210.2	48	–
Mescaline-NBOMe	1354632–01-1	C_19_H_25_NO_4_	2.52	331.9 > 315.1	12	331.9 > 121.1	19	25C-NBOMe D3
3,4-DMA-NBOMe	2748343–73-7	C_19_H_25_NO_3_	2.79	315.9 > 121.1	18	315.9 > 91.2	36	25C-NBOMe D3
DOI	42203–78-1	C_11_H_16_INO_2_	2.97	321.7 > 305.2	13	321.7 > 277.2	20	LSD D3
2C-P	207740–22-5	C_13_H_21_NO_2_	3.74	223.9 > 207.2	12	223.9 > 192.2	19	LSD D3
25H-NBOMe	919797–16-3	C_18_H_23_NO_3_	5.70	301.9 > 121.1	19	301.9 > 91.2	43	25C-NBOMe D3
25N-NBOMe	1354632–03-3	C_18_H_22_N_2_O_5_	5.94	346.9 > 121.1	18	346.9 > 91.2	37	25C-NBOMe D3
25C-NBOH	1391488–16-6	C_17_H_20_ClNO_3_	6.37	321.8 > 216.1	14	321.8 > 199.1	14	25C-NBOMe D3
4-MMA-NBOMe	2703178–01-0	C_19_H_25_NO	6.83	283.9 > 121.1	19	283.9 > 91.2	37	25C-NBOMe D3
4-MA-NBOMe	2749298–47-1	C_18_H_23_NO	7.31	270.0 > 121.1	16	270.0 > 91.2	35	25C-NBOMe D3
25C-NBF	1539266–21-1	C_17_H_19_ClFNO_2_	7.51	323.9 > 199.2	18	323.9 > 184.1	27	25C-NBOMe D3
25B-NBOH	1335331–46-8	C_17_H_20_BrNO_3_	7.73	367.8 > 262.1	15	367.8 > 245.0	21	25C-NBOMe D3
30C-NBOMe	1445574–98-0	C_20_H_26_ClNO_5_	7.76	395.9 > 181.2	20	395.9 > 148.1	41	25C-NBOMe D3
25B-NBF	1539266–17-5	C_17_H_19_BrFNO_2_	8.38	369.9 > 245.2	19	369.9 > 230.1	28	25C-NBOMe D3
25D-NBOMe	1354632–02-2	C_19_H_25_NO_3_	8.46	316.0 > 121.1	19	316.0 > 91.2	36	25C-NBOMe D3
25C-NBOMe	1539266–19-7	C_18_H_22_ClNO_3_	8.51	335.9 > 121.1	18	335.9 > 91.2	36	25C-NBOMe D3
25C-NBOMe D3 (IS)	67–56-1	C_18_H_19_D_3_ClNO_3_	8.52	338.9 > 124.2	19	338.9 > 92.2	40	–
25 T-NBOMe	1539266–47-1	C_19_H_25_NO_3_S	8.86	347.9 > 121.1	20	347.9 > 91.2	37	25C-NBOMe D3
25I-NBOH	919797–20-9	C_17_H_20_INO_3_	9.06	413.8 > 308.1	16	413.8 > 291.2	22	25C-NBOMe D3
4EA-NBOMe	2749282–75-3	C_19_H_25_NO	9.10	283.9 > 121.1	17	283.9 > 91.2	36	25C-NBOMe D3
25B-NBOMe	1026511–90-9	C_18_H_22_BrNO_3_	9.20	379.8 > 121.1	20	379.8 > 91.2	38	25C-NBOMe D3
25I-NBF	919797–21-0	C_17_H_19_FINO_2_	9.55	415.8 > 291.2	20	415.8 > 275.9	30	25C-NBOMe D3
25E-NBOMe	1354632–14-6	C_20_H_27_NO_3_	9.63	329.9 > 121.1	20	329.9 > 91.2	37	25C-NBOMe D3
25I-NBOMe	1043868–97-8	C_18_H_22_INO_3_	10.20	427.9 > 121.1	20	427.9 > 91.0	36	25C-NBOMe D3
25P-NBOMe	1391489–07-8	C_21_H_29_NO_3_	10.63	343.9 > 121.1	21	343.9 > 91.2	38	25C-NBOMe D3
25T4-NBOMe	1566571–73-0	C_21_H_29_NO_3_S	10.81	375.9 > 121.1	21	375.9 > 91.1	39	25C-NBOMe D3

*CE* collision energy, *IS* internal standard, *MRM* multiple reaction monitoring, *RT* retention time

MEPS of oral fluid samples was performed using a SGE Analytical Science (Victoria, Australia) eVol XR digitally controlled positive displacement dispensing system, with a 100-μL syringe (22-gauge needle, 0.72 mm outside diameter, and 55.5 mm length) and an octadecyl (C18) packed sorbent (4 mg silica with 45 μm mean particle size).

A J.P. Selecta, S.A (Barcelona, Spain) microcentrifuge was employed to centrifuge oral fluid samples. Nylon and polytetrafluoroethylene (PTFE) syringe filters (25 mm × 0.22 µm), sodium chloride, and buffer constituents were obtained from Scharlab (Barcelona, Spain). Buffer solutions were prepared at 0.1 and 1.0 M with sodium acetate (pH 5.0), dipotassium hydrogen phosphate (pH 7.0), and sodium carbonate (pH 9.0) salts. Methanol and formic acid for LC–MS and 150-µL conical glass inserts were also obtained from Scharlab.

Blank oral fluid samples were obtained from volunteers who indicated that they had not consumed psychoactive substances, following the ethical guidelines established by the University of Valencia (H1454687358321—drug analysis in biofluids). The volunteers consented to the use of their biological samples in this study, and the data was anonymized to maintain confidentiality.

Field samples were stored in 1.5-mL polypropylene Eppendorf tubes at − 18 °C until analysis. Recovery studies were performed using a pooled sample obtained from blank oral fluids taken from 8 users.

### Extraction of oral fluid samples

A volume of 900 μL sample was spiked with 10 μL of internal standard solution at 1.8 mg L^−1^ in methanol, and the pH was adjusted to 7.0 by adding 100 μL phosphate buffer (pH 7.0, 1 M). Then, the samples were centrifuged at 14,000 rpm for 3 min and filtered with 0.22-µm PTFE syringe filters to remove solids that may clog the syringe.

The extraction of synthetic hallucinogens from oral fluid samples was carried out by MEPS using a C18 sorbent and a plunger speed of 10 μL s^−1^. The sorbent was previously conditioned with 100 μL methanol and 100 μL deionized water. One hundred microlitres of sample was placed in a 150-µL conical glass insert and loaded using three charge/discharge MEPS cycles. The sorbent was washed with 100 μL of deionized water and dried with two charge/discharge MEPS cycles of 100 μL air. Finally, the synthetic hallucinogens were eluted with 50 μL methanol, using a single charge/discharge cycle.

### LC–MS/MS determination

The analysis of synthetic hallucinogens was performed using an Agilent (Palo Alto, CA, USA) 1100 LC, which contained a G1311A quaternary pump, a G1322A vacuum degasser, and a G1329A autosampler, coupled to a Finnigan (Waltham, MA, USA) TSQ Quantum Ultra mass spectrometer system, and a Varian (Palo Alto, CA, USA) PLRP-S 300 Å (150.0 × 1.0 mm, 5 μm) column. Chromatographic separation was performed by the injection of 5 μL of extract using a 0.2-mL min^−1^ mobile phase flow. The mobile phase consisted of 0.1% formic acid in water (A) and 0.1% formic acid in methanol (B), using a gradient programme from 40 to 95% B for 10 min, keeping 95% for 5 min, and returning to initial conditions for 5 min.

MS–MS acquisitions were conducted in positive ion mode using an electrospray ionization (ESI) source. Nitrogen was used as sheath gas (10 psi) and auxiliary gas (15 psi) to assist with nebulization and desolvation. Spray voltage was set at 3000 V and capillary temperature was maintained at 280 °C. Argon was employed as collision gas for collision-induced dissociation (CID) at a pressure of 1.0 mTorr. Quantification was performed using multiple reaction monitoring (MRM) of the transitions shown in Table 1, with a dwell time of 150 ms per transition. Mass calibration was performed by infusing Pierce triple quadrupole calibration solution, obtained from Thermo Fisher Scientific (Waltham, MA, USA), using a Hamilton 500-µL microsyringe at a flow rate of 20 μL min^−1^. Calibrations, data acquisition, and processing were performed using TSQ Series 2.3 SP3 and Xcalibur software from Thermo Fisher Scientific.

Internal standard calibration was employed using standards of the studied synthetic hallucinogens, from 5 to 100 μg L^−1^ (*n* = 6) prepared in methanol, containing 20 μg L^−1^ deuterated internal standards. MEPS extracts were adequately diluted with methanol in case the concentration exceeded the upper range of the calibration line. Statistical treatment of data was carried out using Microsoft Excel software.

## Results and discussion

### LC–MS/MS analytical features

Tandem mass spectrometry conditions, SRM transitions, and collision energy were optimized for the determination of the studied synthetic hallucinogens, by infusing individual standard solutions at 10 µg L^−1^ in methanol, in the same conditions as the MS–MS calibration solution. Table 1 summarizes the MRM transitions and collision energies for all evaluated compounds. Internal standard calibration was employed to improve the reproducibility of the whole procedure. Thus, the most appropriate internal standard was selected for each studied compound, based on similarities in their molecular structures and retention times. Thus, LSD D3 was employed in the calibration of LSD, DOC, DOI, 2C-C, and 2C-P, while 25C-NBOMe D3 was employed in the calibration of NBOMe, NBOH, and NBF derivatives.

Chromatographic conditions were adjusted to obtain an adequate separation with high resolution for all the studied compounds. Several reverse-phase columns were evaluated from different manufacturers and dimensions, with the Varian PLRP-S 300 Å (150.0 × 1.0 mm, 5 μm) column being selected due to its adequate separation of the studied compounds with retention times from 1.5 to 10.8 min. The total analysis time was 20 min, including a cleaning step with 95% mobile phase B and a returning step to initial conditions.

### Evaluation of extraction parameters

An evaluation of the main operational parameters of MEPS was carried out to obtain the highest extraction efficiency of the evaluated synthetic hallucinogens from oral fluid samples. Thus, the sample pH and ionic strength, the number of extraction cycles, the number of elution cycles, and the nature of the elution solvent were evaluated by a monoparametric study, where the effect of each single variable was evaluated while keeping the other variables constant.

Regarding the type of the sorbent, a limited number of materials are commercially available for MEPS cartridges, such as C2, C8, C18, silica-based, and ion exchangers. Among them, C18 was selected as the most adequate sorbent due to its wide use in forensic drug analysis [41] and its good retention capacity and high recoveries in the extraction of hallucinogens from serum [42] and NBOMe derivatives from plasma [15].

The reported pH values of saliva range from 6.2 to 7.6 [43]; however, oral fluids show a wider range of pH depending on the stimulation state, recent consumption of food or drink, and microbial ecosystem activity. Thus, the effect of sample pH was assessed by the analysis of a 50-μg L^−1^ standard solution of target analytes prepared in different 0.1 M buffers that covered pH values of 5.0, 7.0, and 9.0. C18 sorbent was conditioned with 100 μL methanol and 100 μL deionized water, 100 μL spiked buffer solution was loaded using five charge/discharge extraction cycles, and after a washing step with 100 μL deionized water, the extracted analytes were eluted with 100 μL methanol using five single charge/discharge cycles. A volume of 10 μL internal standard solution at 200 µg L^−1^ in methanol was added to the final extract in order to compensate for LC–MS/MS variations, but not consider its C18 extraction.

**Fig. 1:**
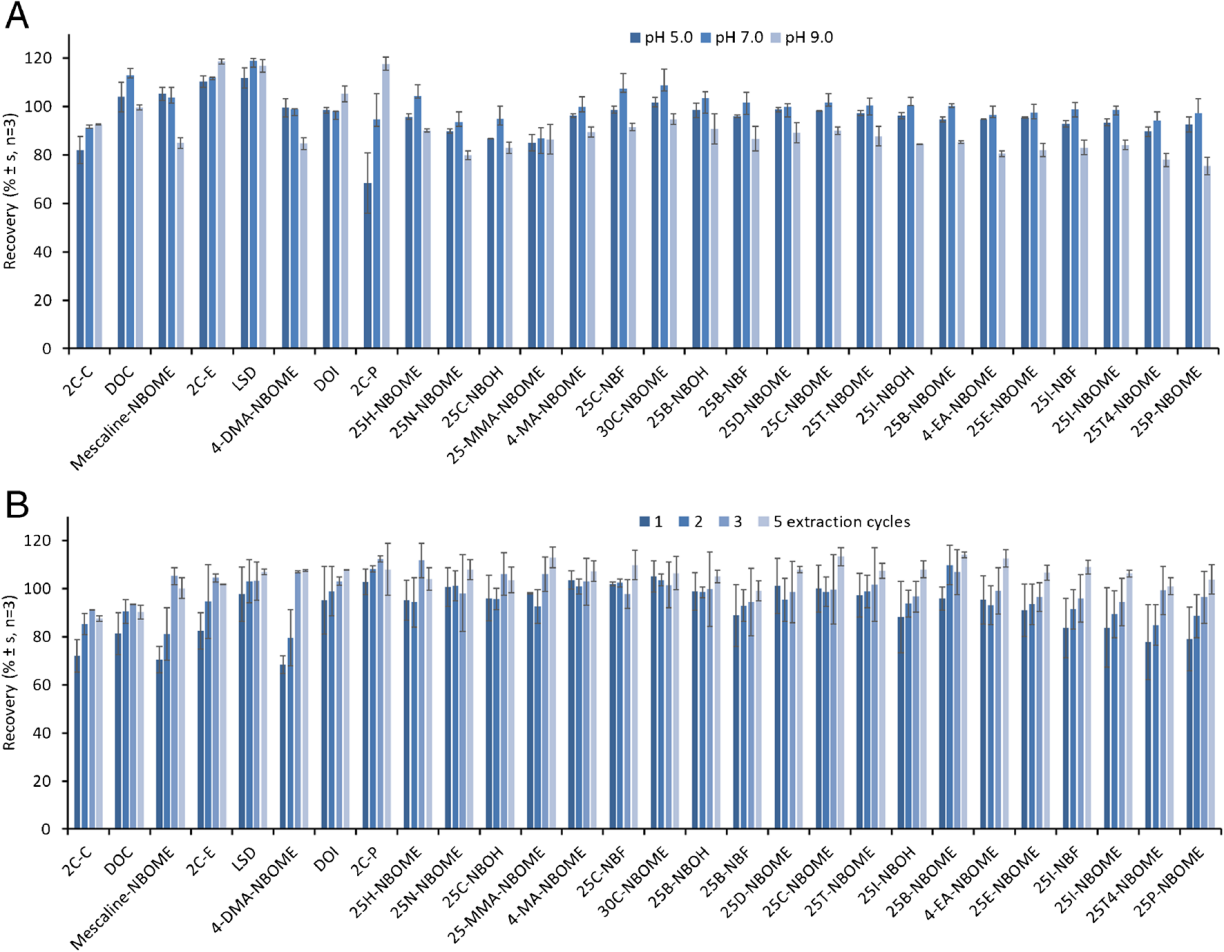
Effect of pH (**A**) and number of extraction cycles (**B**) on the microextraction by packed sorbent of 50 µg L.^−1^ studied synthetic hallucinogens in water (see text for details)

Figure 1A shows the obtained recovery values, indicating a minor influence with sample pH. Extraction at pH 5.0 and 7.0 showed similar recoveries for the studied analytes, ranging from 82 to 112% and from 87 to 119%, respectively. However, recoveries decreased an average of 11% in the extraction of the studied analytes at basic pH (9.0), except for 2C-P that clearly increased its recovery with pH values. As basic amines, the studied compounds are expected to have a pK_a_ value higher than 8.5. Estimated pK_a_ values can be found in literature for some compounds like LSD (7.8), 25B-NBOMe (9.1), 25H-NBOME (9.1), and25H-NBOH (10.4) [44–46], but, to our knowledge no experimental pK_a_ values have been reported yet. Thus, in spite of the fact that the studied compounds were expected to present a positive charge at pH levels lower than 7–8, the obtained recoveries at these pHs were almost quantitative, indicating the existence of non-polar interactions with the C18 sorbent. Thus, the sample pH was adjusted to 7.0 for further experiments, using a phosphate buffer, to obtain the highest extraction efficacy for most of the evaluated substances.

The effect of ionic strength over the recovery was also studied by the addition of a very high content of sodium chloride (5% w/v) to the pH 7.0 phosphate buffer solution spiked with the studied analytes at 50 μg L^−1^. No significant differences were observed over the obtained recoveries, with average variations lower than 5%. Thus, the ionic strength of oral fluid samples is expected to have no effect over the MEPS of the studied compounds.

The number of charge/discharge cycles in the extraction step was evaluated using the previous procedure with 100 µL of a pH 7.0 buffer spiked with 50 µg L^−1^ analytes and 1, 2, 3, and 5 extraction cycles. Figure 1B shows that the use of a single charge/discharge cycle which provided quantitative recoveries from most of the studied compounds. However, 3 cycles are required to obtain recoveries higher than 90% for 2C-C, DOC, mescaline-NBOME, 2C-E, 4-DMA-NBOME, 25I-NBF, 25I-NBOME, 25T4-NBOE, and 25P-NBOME. Thus, 3 charge/discharge cycles were selected to quantitatively extract target analytes from oral fluid samples in the lowest analysis time.

**Fig. 2:**
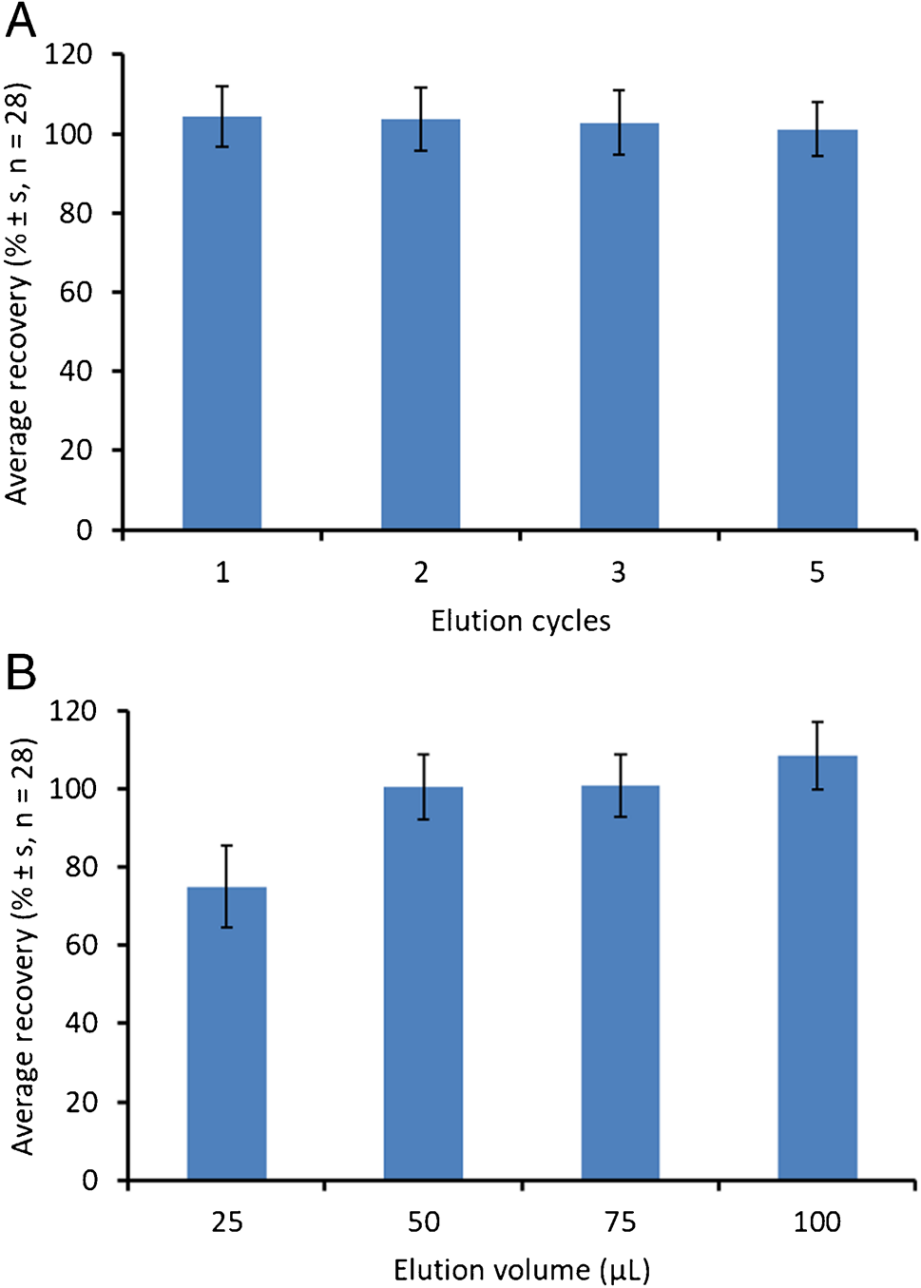
Effect of number of elution cycles (**A**) and elution volume (**B**) on the microextraction by packed sorbent of 50 µg L.^−1^ studied hallucinogens in water (see text for details)

Regarding the elution step, methanol was selected as solvent due to its high elution strength in reverse-phase applications. The number of charge/discharge cycles in the elution step was also evaluated from 1 to 5 cycles using 100 µL methanol. As it can be seen in Fig. 2A, a single charge/discharge cycle was enough to elute the studied analytes from MEPS sorbent (average recoveries from 93 to 110%) and it was selected for further experiments. No other elution solvents were evaluated due to the high recoveries obtained.

The elution volume was reduced to increase the pre-concentration factor of the proposed MEPS procedure, using 75, 50, and 25 µL methanol in a single charge/discharge cycle. Figure 2B shows the obtained recoveries. The use of 25 µL solvent provided an average recovery of 75 ± 11%, with individual recoveries ranging from 48% for 25P-NBOME to 98% for LSD. Nevertheless, the use of elution volumes higher than 50 µL methanol provided quantitative recoveries for the studied analytes with values ranging from 84 to 115%. Thus, 50 µL methanol was selected as elution volume, providing a pre-concentration factor of 2.

### Extraction of oral fluids

**Fig. 3:**
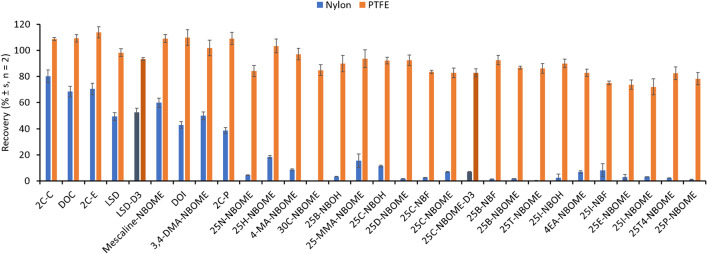
Recoveries obtained for the filtration of a 50-µg L^−1^ hallucinogen aqueous solution with 0.22-µm nylon and PTFE syringe filters

Oral fluids are heterogeneous samples composed mainly of a liquid matrix that may contain solid residues. Thus, solid particles must be removed from oral fluid samples before the MEPS procedure to avoid any syringe clogging. Thus, as mentioned above, a 14,000-rpm centrifugation step was proposed to separate solid particles from samples, followed by a filtration step with 0.22-µm syringe filters. The nature of the filter membrane has a high relevance when aqueous solutions of non-polar compounds are filtered, because some retention can be observed. Thus, syringe filters with different polarities, such as nylon (hydrophilic) and PTFE (hydrophobic), were employed for the filtration of a 50-µg L^−1^ solution of the studied analytes in pH 7.0 phosphate buffer. Figure 3 shows the obtained recoveries, indicating that nylon filters must be completely avoided due to the high retention of most of the evaluated synthetic hallucinogens, probably due to hydrogen bond interactions with nylon polymer. Similar effects have been observed in other studies that involved the filtering of drugs and pharmaceuticals from wastewater and surface waters [47, 48]. Thus, nylon filters provided insignificant recoveries (from 4 to 18%) for most of the NBOME derivatives, and low recoveries from 39 to 80% were obtained for 2C-C, DOC, 2C-E, LSD, LSD-D3, mescaline-NBOME, DOI, 3,4-DMA-NBOME, and 2C-P. However, the use of PTFE filters, without hydrogen bond interactions, provided adequate recoveries between 72 and 114% for all the evaluated analytes and were proposed for the filtration of oral fluid samples after the centrifugation step.

### Method performance verification

The analytical performance of the proposed determination of synthetic hallucinogens in oral fluid samples by MEPS and LC–MS/MS was evaluated by using the following parameters: linearity, limit of detection (LOD), limit of quantification (LOQ), precision, accuracy, and matrix effect. The obtained analytical figures of merit are shown in Table 2.

**Table 2:** Analytical features for hallucinogen determination in oral fluids by MEPS and LC–MS/MS

Analyte	*R*^2^	LOD (µg L^−1^)	LOQ (µg L^−1^)	RSD (%)	Recovery (% ± *s*, *n* = 3)		
					20 μg L^−1^	50 μg L^−1^	100 μg L^−1^
2C-C	0.998	0.80	2.66	6.2	108 ± 3	85 ± 2	104 ± 2
DOC	0.993	1.22	4.06	9.3	123 ± 5	98 ± 4	105 ± 3
2C-E	0.992	0.34	1.12	2.3	123 ± 2	105 ± 4	119 ± 3
LSD	0.997	0.74	2.47	5.3	129 ± 5	108 ± 3	118 ± 6
Mescaline-NBOMe	0.990	0.78	2.61	5.4	126 ± 5	120 ± 4	118 ± 4
3,4-DMA-NBOMe	0.993	0.21	0.69	2.8	127 ± 1	123 ± 2	118 ± 2
DOI	0.990	0.58	1.94	4.3	113 ± 6	103 ± 8	118 ± 3
2C-P	0.990	0.90	2.99	6.5	117 ± 6	104 ± 3	116 ± 2
25H-NBOMe	0.991	0.27	0.88	1.8	124 ± 6	104 ± 9	119 ± 2
25N-NBOMe	0.994	0.29	0.96	4.7	129 ± 5	111 ± 2	119 ± 6
25C-NBOH	0.998	0.44	1.45	5.0	112 ± 6	106 ± 2	109 ± 4
25-MMA-NBOMe	0.993	1.16	3.85	8.0	124 ± 3	106 ± 4	121 ± 2
4-MA-NBOMe	0.993	0.24	0.81	3.4	119 ± 4	105 ± 4	105 ± 4
25C-NBF	0.996	0.09	0.29	0.9	108 ± 5	102 ± 4	104 ± 3
25B-NBOH	0.995	0.41	1.36	3.1	124 ± 4	102 ± 2	111 ± 2
30C-NBOMe	0.996	0.25	0.84	3.4	80 ± 7	110 ± 3	121 ± 1
25B-NBF	0.996	0.87	2.89	6.1	95 ± 8	103 ± 8	113 ± 2
25D-NBOMe	0.993	0.29	0.96	2.0	121 ± 5	113 ± 2	119 ± 6
25C-NBOMe	0.994	0.64	2.14	4.3	122 ± 5	119 ± 4	115 ± 9
25 T-NBOMe	0.993	0.67	2.23	4.8	125 ± 4	115 ± 3	123 ± 4
25I-NBOH	0.997	0.32	1.07	3.7	90 ± 10	109 ± 1	115 ± 2
4EA-NBOMe	0.991	0.27	0.89	1.9	108 ± 8	100 ± 2	114 ± 3
25B-NBOMe	0.995	0.45	1.48	3.1	87 ± 9	113 ± 5	119 ± 3
25I-NBF	0.995	0.67	2.09	4.2	95 ± 9	110 ± 3	118 ± 2
25E-NBOMe	0.994	0.41	1.36	2.9	95 ± 10	109 ± 4	119 ± 3
25I-NBOMe	0.994	0.97	3.22	7.0	90 ± 9	110 ± 1	121 ± 5
25P-NBOMe	0.993	0.30	0.99	2.1	108 ± 2	88 ± 9	104 ± 9
25T4-NBOMe	0.993	0.40	1.34	3.0	110 ± 3	90 ± 5	110 ± 8

*LOD* limit of detection, *LOQ* limit of quantification, *RSD* relative standard deviation

Linearity was evaluated from 5 to 100 μg L^−1^, providing a deviation of residuals of less than 5% and coefficients of determination (*R*^2^) higher than 0.990 for all the evaluated analytes. The precision of LC–MS/MS measurements was evaluated as the relative standard deviation (RSD) of a 5-μg L^−1^ standard solution of analytes (*n* = 5), the values being between 0.9 and 9.3%. LOD and LOQ values were calculated as three and ten times the standard deviation of a 5-μg L^−1^ standard solution of analytes (*n* = 5) divided by the slope of the calibration curve, and considering a pre-concentration factor equal to 2. LODs ranged from 0.09 to 1.22 µg L^−1^, while LOQs ranged from 0.29 to 4.06 µg L^−1^ for 25C-NBF and DOC, respectively.

To our knowledge, no certified reference samples were available containing NBOME, NBOH, or NBF derivatives. Thus, the accuracy and precision of the method was assessed as the obtained recovery and RSD values for the analysis of three quality control (QC) samples prepared using different blank oral fluid samples spiked at three concentration levels (low, medium, and high) of the studied analytes. The obtained recoveries were quantitative for most of the evaluated compounds (Table 2), with values ranging from 80 to 129% for low QC (20 μg L^−1^), from 85 to 123% for medium QC (50 μg L^−1^), and from 104 to 123% for high QC (100 μg L^−1^). The acceptance criteria for accuracy of the American Academy of Forensic Sciences for method validation in forensic toxicology are recovery values within the range of 80–120% [49]. In this study, most of the evaluated synthetic hallucinogens fulfil this criterion and only a few exceptions were observed for a reduced number of compounds, especially in the low concentration level (20 μg L^−1^) with recovery values no higher than 130%. Thus, the obtained recoveries for the MEPS of synthetic hallucinogens from oral fluids were considered quantitative. The precision of the method was very high, with RSD values of QC samples from 1 to 9%. Consequently, the proposed MEPS LC–MS/MS methodology provided accurate and precise results for the determination of synthetic hallucinogens in oral fluids.

**Fig. 4:**
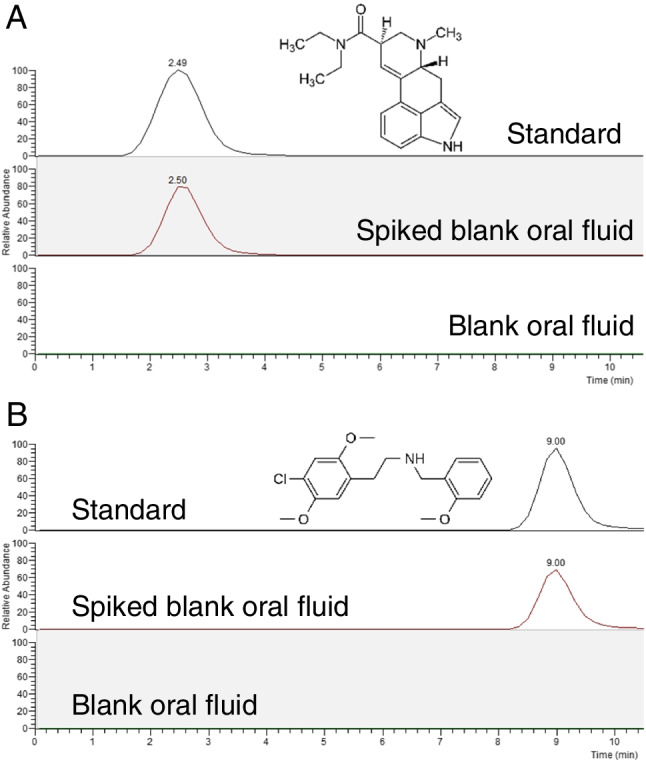
LC–MS/MS multiple reaction monitoring chromatograms for LSD (**A**) and 25C-NBOME (**B**) obtained for a blank oral fluid sample, a blank oral fluid sample spiked with the studied synthetic hallucinogens at 25 µg L^−1^ concentration level, and a 50-µg L^−1^ standard solution

Figure 4 shows the LSD and 25C-NBOME MRM chromatograms obtained for a 50-μg L^−1^ standard in methanol, a blank oral fluid sample, and a blank oral fluid sample spiked at 25 μg L^−1^ of studied analytes. As it can be seen, the obtained chromatograms for the blank oral fluid sample extracted by MEPS provided very clean extracts with no interferent signals, the combination of MEPS and LC–MS/MS being a very selective approach. Chromatograms obtained for the spiked sample allow for a sensitive determination of the studied synthetic hallucinogens in oral fluids.

The determination of analyte traces in complex matrices by LC–MS/MS is commonly affected by ionization suppression/enhancement processes due to the matrix effect. Thus, the matrix effect (ME) was calculated for the direct LC–MS/MS analysis of a blank oral fluid sample spiked with 50 µg L^−1^ of the studied analytes and its internal standards, and by the analysis of the same spiked sample after a 1:1 dilution with methanol and after the MEPS using the proposed procedure. ME was calculated as the average peak areas obtained for the spiked blank oral fluid samples, after the aforementioned treatments, divided by the average peak areas of a 50-µg L^−1^ standard in methanol multiplied by 100, as shown in Eq. 1 [50].

1 $$\mathrm{ME}\left(\%\right)=\frac{{\overline A}_{\mathrm{oral}\;\mathrm{fluid}}-{\overline A}_{\mathrm{standard}}}{{\overline A}_{\mathrm{standard}}}100$$

The acceptance criterion of the American Academy of Forensic Sciences for method validation in forensic toxicology was a matrix effect lower than 20% [49]. Table 3 shows the obtained ME values for each studied analyte for the three different oral fluid treatments evaluated. As it can be seen, direct analysis of oral fluids provided a considerable matrix enhancement with an average ME value of 51 ± 13%. A 1:1 dilution of matrix reduced matrix enhancement, but sensitivity was compromised and ME was still significative with average values of 20.8 ± 17%. However, the proposed MEPS methodology provided an average ME value of 14.5%, with individual values lower than 20% for all the evaluated compounds, except for 25-MMA-NBOME, 30C-NBOME, and 25 T-NBOME that slightly exceed this value till 22%. Thus, MEPS with C18 sorbents shows a good capacity to remove sample matrix interferents and matrix matched standards were not required for LC–MS/MS calibration, being performed by standards prepared in methanol. Moreover, the MEPS procedure provided a 2-times pre-concentration factor that considerably improved the sensitivity of the method.

**Table 3:** Matrix effect calculated for a blank oral fluid sample spiked with 50 µg L^−1^ of the studied analytes analysed directly by LC–MS/MS, after a 1:1 dilution with methanol, and after MEPS developed procedure

Analyte	ME (%)		
	Direct	1:1 dilution	MEPS
2C-C	49.8	44.1	4.3
DOC	83.3	47.1	5.4
2C-E	75.7	67.4	17.7
LSD	39.1	34.6	17.3
Mescaline-NBOMe	70.9	40.2	22.3
3,4-DMA-NBOMe	34.9	40.9	16.9
DOI	58.5	15.4	18.3
2C-P	61.6	52.8	16.5
25H-NBOMe	59.6	17.3	17.2
25N-NBOMe	54.1	20.5	16.2
25C-NBOH	59.8	16.3	9.5
25-MMA-NBOMe	52.8	22.8	20.4
4-MA-NBOMe	54.5	16.8	5.1
25C-NBF	46.6	9.4	4.2
25B-NBOH	46.2	5.5	11.0
30C-NBOMe	60.0	22.0	20.8
25B-NBF	34.6	5.0	13.0
25D-NBOMe	51.9	15.2	18.7
25C-NBOMe	51.5	18.4	15.4
25 T-NBOMe	51.9	15.0	21.9
25I-NBOH	37.4	12.7	15.2
4EA-NBOMe	44.2	13.1	14.2
25B-NBOMe	49.0	15.4	17.8
25I-NBF	32.0	6.6	18.4
25E-NBOMe	41.7	9.9	16.2
25I-NBOMe	43.0	9.6	19.6
25P-NBOMe	45.8	-4.7	3.9
25T4-NBOMe	33.8	-6.7	10.1
Average	50.9	20.8	14.5
Standard deviation	12.6	17.4	5.7
Maximum	83.3	67.4	22.3
Minimum	32.0	− 6.7	3.9
Pre-concentration factor	1.0	0.5	2.0

## Conclusions

Synthetic hallucinogens are threatening psychoactive substances that are nowadays slightly monitored in spite of the numerous fatal consequences highlighted by case reports. In this study, an analytical procedure based on MEPS coupled to LC–MS/MS has been developed for the simultaneous determination of 28 synthetic hallucinogenic substances in oral fluid samples. In our knowledge, this is the first precedent in the analysis of a wide range of NBOME derivates and other synthetic hallucinogen substances from oral fluid samples. Experimental conditions for MEPS of the target analytes had been adjusted in order to obtain quantitative extraction yields. The obtained recoveries ranged from 80 to 129% with a high sensitivity and with LOD values from 0.09 to 1.22 μg L^−1^. The analytical performance of the proposed procedure was evaluated, showing satisfactory results for linearity, sensitivity, precision, and accuracy for all analytes. The use of a semiautomatic extraction procedure based on MEPS has shown a high potential to perform routine analysis of a high number of samples, with a reduced solvent consumption and environmental impact, in the identification of recent consumption of hallucinogens and driving under the influence.

## Declarations

Blank oral fluid samples were obtained following the ethical guidelines established by the University of Valencia (H1454687358321—drug analysis in biofluids).
